# Influenza D Virus Circulation in Cattle and Swine, Luxembourg, 2012–2016

**DOI:** 10.3201/eid2407.171937

**Published:** 2018-07

**Authors:** Chantal J. Snoeck, Justine Oliva, Maude Pauly, Serge Losch, Félix Wildschutz, Claude P. Muller, Judith M. Hübschen, Mariette F. Ducatez

**Affiliations:** Luxembourg Institute of Health, Esch-sur-Alzette, Luxembourg (C.J. Snoeck, M. Pauly, C.P. Muller, J.M. Hübschen);; Interactions Hôtes Agents Pathogènes, Université de Toulouse, INRA-ENVT, Toulouse, France (J. Oliva, M.F. Ducatez);; Administration des Services Vétérinaires de l’Etat, Ministère de l'Agriculture, Dudelange, Luxembourg (S. Losch, F. Wildschutz);; Laboratoire National de Santé, Dudelange (C.P. Muller)

**Keywords:** influenza D virus, seroprevalence, serology, cattle, pig, swine, Luxembourg, influenza, viruses

## Abstract

We detected antibodies against influenza D in 80.2% of the cattle sampled in Luxembourg in 2016, suggesting widespread virus circulation throughout the country. In swine, seroprevalence of influenza D was low but increased from 0% to 5.9% from 2012 to 2014–2015.

Influenza D virus (IDV), a new orthomyxovirus distantly related to influenza C virus, was described in pigs with respiratory symptoms in 2011 (*1*). Although mild symptoms only were reported in experimental pig and calf infections, the virus has been implicated in bovine respiratory disease complex (*1**–**3*). Cattle are currently considered the main host of the virus, but other livestock species are also susceptible (*4*). In Europe, IDV circulation has been reported in France (*5*), Italy (*6**,**7*), and Ireland (*8*). Recent serosurveys in Italy showed extremely high seroprevalence rates in cattle (92.4% seropositive) (*9*) and a low but increasing seroprevalence in swine, from 0.6% in 2009 to 11.7% in 2015 (*7*). We investigated the presence of IDV in cattle and swine farms in Luxembourg.

In 2016, we collected serum samples from 450 asymptomatic cattle from 44 farms throughout Luxembourg (Figure, panel A; Technical Appendix). We screened the samples for IDV antibodies by using hemagglutination inhibition (HI) assays. We also screened serum samples collected from pigs at 2 slaughterhouses in 2012 (n = 258, 27 farms) and 2014–2015 (n = 287, 29 farms). Because HI titers as low as 20 were measured in farms with demonstrated influenza D circulation (*7*), we considered HI titers >20 positive. In addition, we screened nasal swab specimens from asymptomatic pigs sampled at slaughter in 2009 (n = 232, 56 farms) and 2014–2015 (n = 427, 36 farms) by real-time reverse transcription PCR (*1*). No cattle samples were available for molecular screening.

**Figure:**
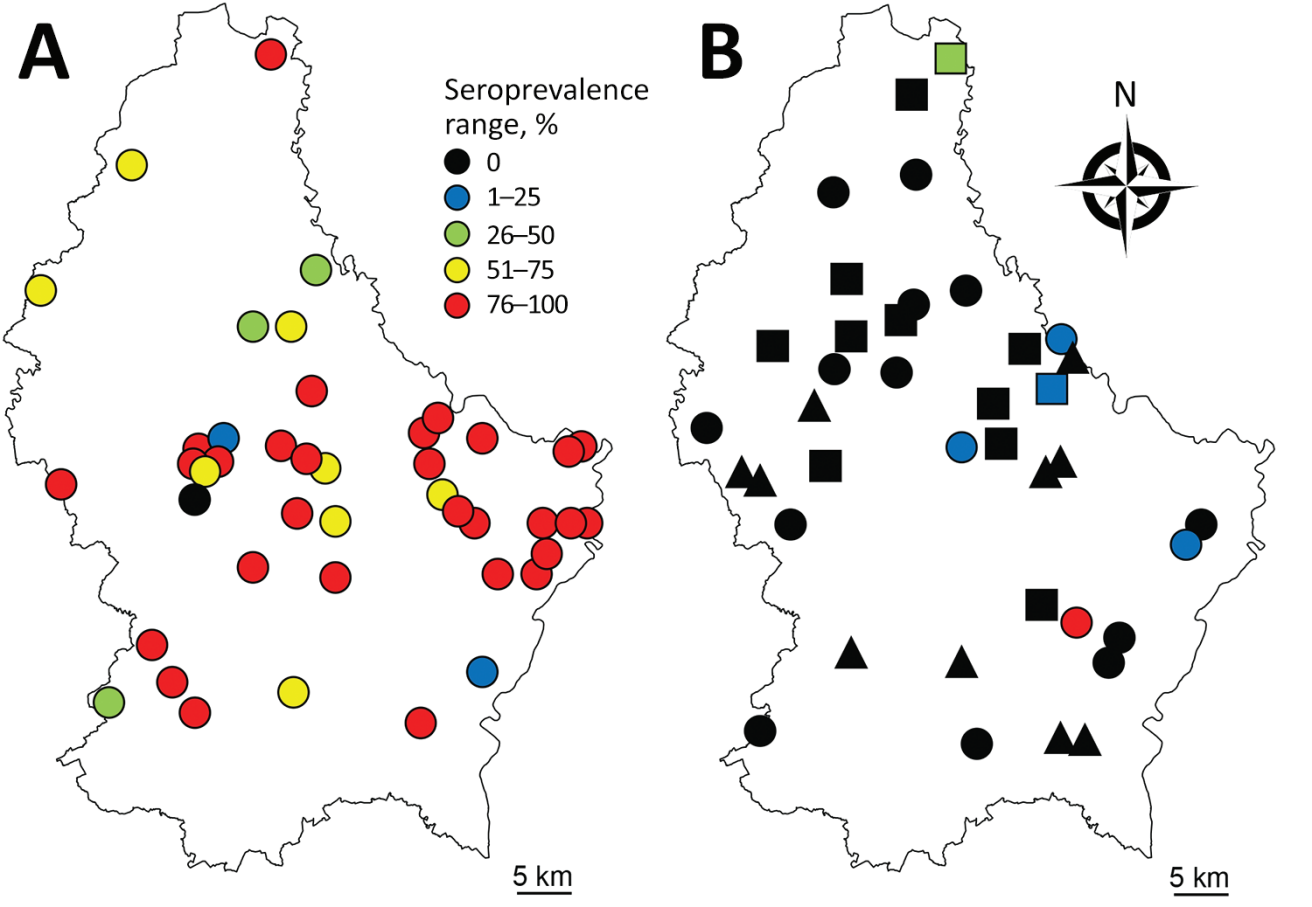
Within-farm seroprevalence range in A) cattle herds (n = 44) sampled in 2016 and B) swine herds sampled in 2012 (n = 10, triangles), 2014–2015 (n = 12, squares) or both (n = 17, circles).

We found an overall seroprevalence of 80.2% in cattle (361/450; HI titer range 20–1,280) (online Technical Appendix Figure); 97.7% of herds (43/44) had >1 seropositive animal. Average within-farm seroprevalence was 83.0% (range 20%–100%; Figure, panel A). These results suggested that IDV affects most animals in nearly all farms (Figure, panel A). All animals were much older than 6 months (average 70.5 mo, range 23–209 mo), so it is unlikely that the antibodies were maternally derived (*10*). The median age of seropositive animals (61 months) was significantly higher than the median age of seronegative animals (41 months; p<0.001). Seroprevalence was higher in beef cattle (88.0%, 95/108) than in dairy cattle (75.6%, 133/176; meat or dairy production type was not known for 166 animals), but beef cattle were also on average older than dairy cattle. A binary logistic regression model including herd as a random effect and age and production type as fixed effects revealed that only age substantially affected IDV seropositivity. 

Most of the cattle investigated were born in Luxembourg (90%, 405/450), but IDV antibodies were found regardless of country of birth (others were born in Germany, France, Belgium, and Italy). This information demonstrates that our results cannot be explained by importation of seropositive animals alone and that IDV transmission takes place in Luxembourg. Within-herd seroprevalence of cattle herds was similar for herds located near the borders as well as those further inland, suggesting that the virus could also spread to and from the neighboring countries (Belgium, France, and Germany), for example, through cross-border grazing.

In Luxembourg, IDV seroprevalence was low in swine compared with cattle but has increased during recent years (0% in 2012 to 5.9% [17/287] in 2014–2015), as it has in Italy (*7*). We detected seropositive animals in 6/29 (20.7%) swine herds (Figure, panel B). The low virus prevalence from nasal swabs (0% in 2009, 0.7% [3/427] in 2014–2015) and the low viral RNA concentration (9.7–94.5 copies/μL) were not conducive to amplification of genetic material for sequencing. The low levels of virus circulation in pigs shown by seroprevalence data, the absence of symptoms at the time of sampling (*3*), and the short shedding period under experimental infection (*1*) probably contributed to the low detection rates observed in swine nasal swab samples. The IDV RNA–positive nasal swab samples originated from 2 different herds, 1 of which was also seropositive (9/10 pigs with HI titer >20; no samples were available from the second herd). Although we collected all 3 PCR-positive samples on the same day at the same slaughterhouse, it is unlikely that the pigs were infected during their short stay there.

Taken together, our results suggested that IDV circulates widely throughout cattle farms in Luxembourg and can be considered hyperenzootic in the country. Once introduced into a herd, IDV seems to spread very efficiently, given the high within-farm seroprevalence rates. In light of cross-border trade and grazing, the region beyond Luxembourg’s borders may be also hyperenzootic for IDV. Although IDV mainly affects cattle, we detected IDV antibodies in pigs and an increased seroprevalence in pig herds. We are planning systematic serologic and virologic screenings along with epidemiologic surveys to investigate the genetic diversity of IDV strains in Luxembourg, to evaluate the effect of IDV infection on cattle and pig health and productivity, and to study IDV interaction with other pathogens.

Technical Appendix Additional information about influenza D virus circulation in cattle and swine in Luxembourg, 2012–2016.
