# Supplementary material for: Influenza D Virus Circulation in Cattle and Swine, Luxembourg, 2012–2016
Source: Emerg Infect Dis. 2018 Jul;24(7):1388–9. doi: 10.3201/eid2407.171937 (PMC6038750; doi:10.3201/eid2407.171937)
Supplement: Technical Appendix — Additional information about influenza D virus circulation in cattle and swine in Luxembourg, 2012–2016. [file 17-1937-Techapp-s1.pdf]

Article DOI: https://doi.org/10.3201/eid2407.171937

Influenza D Virus Circulation in Cattle and Swine, Luxembourg, 2012–2016

Technical Appendix

Materials and Methods

Sampling Design and Sample Collection

The 2012 annual census performed by the National Institute of Statistics and Economic Studies of the Grand Duchy of Luxembourg indicated there were 1,218 farms rearing ≥20 cattle and 86 farms rearing ≥20 swine in the country. Our study included cattle serum samples (n = 450) from 44 herds collected in 2016 in the framework of infectious bovine rhinotracheitis serologic monitoring. On average, we screened 10.2 animals (range 9–20)/herd for the presence of influenza D antibodies. No information regarding past respiratory disease in the farms was available.

For pigs, we screened an average of 9.6 serum samples (range 1–19/herd, total = 258) from 27 pig farms sampled in 2012. In the 2014–2015 cohort, we collected an average of 9.9 serum samples/herd (range 9–10/herd, total = 287) from 29 pig farms. We collected all pig samples at the time of slaughter and, as with the cattle, no information regarding past respiratory disease in the herd was available.

Given the paucity of between- and within-herd seroprevalence data at the time of study design (*1*–*3*), and the complete lack of information from Europe, we arbitrarily and conservatively set values of between-herd seroprevalence to 10% and within-herd seroprevalence to 30%. According to the formula of Cannon and Roe (1982; cited in (*1*)), screening 10 animals/herd was sufficient to find ≥1 seropositive animal in a seropositive herd if the within-herd seroprevalence is ≥30%, irrespective of herd size (assuming a 90% test sensitivity and a desired certainty of detecting past infection in an infected herd of 95%). Similarly, sampling 44/1,218 cattle herds and 27/86 swine farms was sufficient to find ≥1seropositive herd if between-herd seroprevalence was ≥10%.

We screened nasal swab samples from asymptomatic pigs at slaughter for the presence of influenza D virus. In 2009, we sampled 56 farms and screened 4.1 samples/herd on average (range 1–15/herd, n = 232). In 2014–2015, we collected 11.9 nasal swabs/herd on average from 36 pig farms (range 5–20/herd, n = 427).

Hemagglutination Inhibition (HI) Assays

We screened all samples for the presence of IDV antibodies by HI tests performed as previously described (*2*) using D/bovine/France/5920/2014 viral strain (D/swine/Oklahoma/2011 lineage). We pretreated all serum samples with receptor-destroying enzyme (RDE, DebenDiagnostics, Germany) and hemadsorbed them on horse red blood cells before testing in 2-fold serial dilutions from 1:10 until 1:1280. We expressed antibody titers as the reciprocal of the highest serum dilution that inhibited hemagglutination.

Real-time PCR

Pig nasal swabs were screened by a real-time RT-PCR (*3*) with Quantitect Probe RT-PCR kit (QIAGEN, Venlo, The Netherlands). No fluorescence and no probe degradation was visible in any negative controls. We retested positive samples in triplicate to confirm their positive status and quantified them in a qRT-PCR reaction. We generated a standard curve from 10-fold dilutions of a plasmid quantified by absorbance measurement with NanoDrop ND-1000 (Isogen Life Science, De Meern, The Netherlands).

Statistical Analyses

We performed statistical analyses (Mann-Whitney Rank Sum test, χ^2 test) in SigmaPlot version 12.0 (Systat Software Inc., San Jose, CA, USA). We tested a generalized linear mixed-effect model using binary outcome including herd as random effect and age and production type as fixed effects using the R package “lme4” (*4*) in R Studio.

References

1. De Wit JJ. Detection of infectious bronchitis virus. Avian Pathol. 2000;29:71–93. PubMed http://dx.doi.org/10.1080/03079450094108

2. Salem E, Cook EAJ, Lbacha HA, Oliva J, Awoume F, Aplogan GL, et al. Serologic evidence for influenza C and D virus among ruminants and camelids, Africa, 1991–2015. Emerg Infect Dis. 2017;23:1556–9. PubMed http://dx.doi.org/10.3201/eid2309.170342

3. Hause BM, Ducatez M, Collin EA, Ran Z, Liu R, Sheng Z, et al. Isolation of a novel swine influenza virus from Oklahoma in 2011 which is distantly related to human influenza C viruses. PLoS Pathog. 2013;9:e1003176. PubMed http://dx.doi.org/10.1371/journal.ppat.1003176

4. Bates D, Mächler M, Bolker B, Walker S. Fitting linear mixed-effects models using lme4. J Stat Softw. 2015;67:1–48. http://dx.doi.org/10.18637/jss.v067.i01

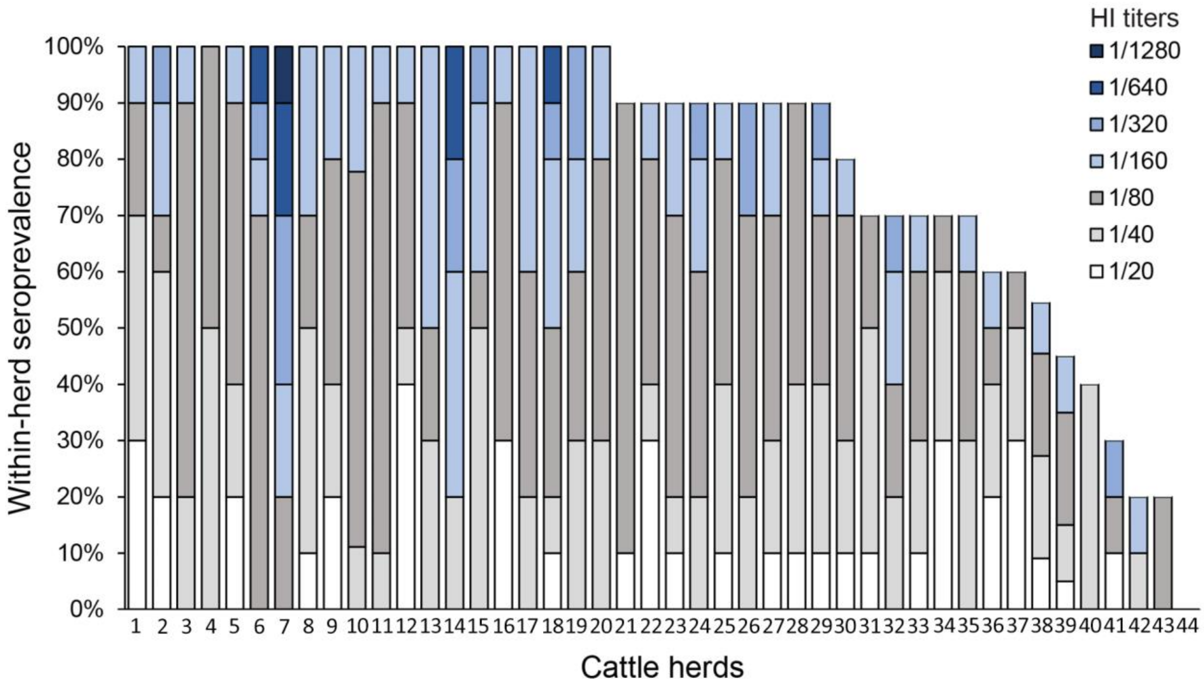

Technical Appendix Figure. Within-herd influenza D seroprevalence of each cattle herd (n = 44) tested and distribution of HI titers of individual animals within each herd (10 animals tested/herd; except for herd #10, n = 9; herd #38, n = 11; herd #39, n = 20).
